# Overcoming cisplatin resistance by mTOR inhibitor in lung cancer

**DOI:** 10.1186/1476-4598-4-25

**Published:** 2005-07-20

**Authors:** Chunjing Wu, Medhi Wangpaichitr, Lynn Feun, Marcus Tien Kuo, Carlos Robles, Theodore Lampidis, Niramol Savaraj

**Affiliations:** 1 Section of Hematology/Oncology, V.A. Medical center, 1201 NW 16^th ^Street, Miami, FL 33125, USA; 2 University of Miami School of Medicine, 1475 NW 12 Ave, Miami, FL 33136, USA; 3 M.D. Anderson Cancer Center, 1515 Holcombe Blvd, Houston, TX. 77030, USA

## Abstract

**Background:**

Cisplatin resistance is complex and involves several different mechanisms. Employing cDNA microarray analysis, we have found that cisplatin resistant cells share the common characteristic of increase in ribosomal proteins and elongation factors. We hypothesize that in order to survive cisplatin treatment, cells have to synthesize DNA repair proteins, antiapoptotic proteins and growth-stimulating proteins. Thus, by blocking the translation of these proteins, one should be able to restore cisplatin sensitivity. We have studied the role of CCI-779, an ester analog of rapamycin which is known to inhibit translation by disabling mTOR, in restoring cisplatin sensitivity in a panel of cisplatin resistant cell lines. We have also determined the role of CCI-779 in P-gp1 and MRP1 mediated resistance.

**Results:**

Our data show that CCI-779 possess antiproliferative effects in both cisplatin sensitive and resistant cell lines, but shows no effect in P-gp1 and MRP1 overexpressing cell lines. Importantly, CCI-779 at 10 ng/ml (less that 10% of the growth inhibitory effect) can increase the growth inhibition of cisplatin by 2.5–6 fold. Moreover, CCI-779 also enhances the apoptotic effect of cisplatin in cisplatin resistant cell lines. In these resistant cells, adding CCI-779 decreases the amount of 4E-BP phosphorylation and p-70S6 kinase phosphorylation as well as lower the amount of elongation factor while cisplatin alone has no effect. However, CCI-779 can only reverse P-gp mediated drug resistance at a higher dose(1 ug/ml).

**Conclusion:**

We conclude that CCI-779 is able to restore cisplatin sensitivity in small cell lung cancer cell lines selected for cisplatin resistance as well as cell lines derived from patients who failed cisplatin. These findings can be further explored for future clinical use. On the other hand, CCI-779 at achievable clinical concentration, has no growth inhibitory effect in P-gp1 or MRP1 overexpressing cells. Furthermore, CCI-779 also appears to be a weak MDR1 reversal agent. Thus, it is not a candidate to use in MDR1 or MRP1 overexpressing cells.

## Introduction

Cisplatin and its analog carboplatin have significant antitumor activity against a wide variety of solid tumors[1]. Furthermore, cisplatin also showed synergistic effect with other chemotherapeutic agents and therefore has been incorporated in many treatment regimens for solid tumors [1]. Like other chemotherapeutic agents, resistance to this drug is inevitable and often occurs after several cycles of treatment. Several laboratories have studied the mechanism(s) of cisplatin resistance in the past decades and many possible mechanism(s) have been identified [2-9]. These resistance mechanism(s) appears to fall into four major categories. The first category involves DNA damage/repair proteins. The second category involves drug retention (increased influx or decreased uptake). The third category involves increased drug inactivation or prevention of drug to reach the DNA target. The fourth category involves growth signaling via different pathways or increase in antiapoptotic protein(s). Nevertheless, it is generally accepted that cisplatin resistance most likely has multiple mechanisms and the mechanism of resistance may differ depending on the cell types. Therefore, overcoming cisplatin resistance may be difficult. We have developed two pairs of cisplatin resistant small cell lung cancer cell lines and three cell lines derived from patients who failed cisplatin, one from small cell lung cancer (SCLC) and two from non small cell lung cancer (NSCLC). Using microarray analysis in our cisplatin resistant cell lines as well as reviewing the available microarray data published in other laboratories, we have found that the there are many gene(s) which are overexpressed in these cisplatin resistant cell lines including those involved in DNA repair, signal transduction, invasion and metastasis, and antiapoptosis {for review see ref. [2]}. While different cisplatin resistant cell lines often overexpressed different genes involved in DNA repairs and/or signal transduction, antiapoptosis, a majority of cisplatin resistant cells overexpress elongation factor alpha and genes which are involved in ribosomal biogenesis. These findings have led us to hypothesize that after DNA damage by cisplatin, the surviving cells have to develop the ability to generate repair proteins and/or survival proteins to prepare for the next insult. Both ribosomal proteins and elongation factor are essential for translational process in protein synthesis. Thus, the common theme to survive cisplatin in these resistant cell lines is to increase these mRNAs. Consequently, if ribosomal proteins and/or elongation factor can be inhibited, one should be able to restore cisplatin sensitivity. It is well known that mTOR (mammalian target of rapamycin) also known as FRAP, RAFT, or RAPT is important in regulating translation of a set of mRNA which encode ribosomal proteins and elongation factor [10-12]. All of these mRNAs possess a sequence of pyrimidines at their extreme 5'end (TOP mRNAs). Therefore, by inhibiting mTOR, one should be able to restore cisplatin sensitivity. In this report, we have investigated the possible role of a known mTOR inhibitor, rapamycin and its ester analog CCI-779 [13,14] in restoring cisplatin sensitivity.

It has been shown that the mTOR inhibitor, rapamycin, can also modulate other forms of drug resistance such as P-gp1 or MDR1 mediated drug resistance[15,16]. MDR1 is a well characterized form of drug resistance which is primarily due to overexpression of an P-gp1 efflux pump [17,18]. This efflux pump belong to ABC (ATP-binding-cassette) transporter superfamily, and is capable of effluxing many different chemotherapeutic agents, hence the term multidrug resistance. The resistance is due to decrease drug accumulation. Rapamycin has been shown to be able to reverse this form of drug resistance by blocking the efflux pump[15]. Another similar form of multidrug resistance which is due to decrease drug accumulation is the MRP1 mediated drug resistance. MRP1 also belongs to the ABC transporter superfamily [19-21], however, this efflux pump most likely transports the glutathione conjugated drug. In this report, we also investigated the role of mTOR inhibitor in these two forms of multidrug resistance.

## Methods

### Cell lines

*SCLC1 *was established from the bone marrow of SCLC patient. SR-2 is the cisplatin resistant variant which was generated by intermittent exposure to cisplatin. The characteristics of these cell lines have been published previously [22]. All cell lines were maintained on RPMI media supplemented with 10% FBS.

*SCLC1R *was generated by exposure of SCLC1 to rhodamine-123 [23]. This cell line overexpresses P-gp and exhibits 16 fold resistance to doxorubicin, 70 fold resistance to vinblastine and 5.3 fold resistance to VP-16, but not to cisplatin.

*SCLC1A *overexpresses MRP1 and was selected by exposure of SCLC1 to doxorubicin [24]. This cell line exhibits 33 fold resistance to doxorubicin, 10 fold resistance to VP-16 and 42 fold resistance to vinblastine.

*SCLCB *was established from the supraclavicular lymph node of a SCLC patient. The characteristics of this cell line were previously published [25]. SCLCBC was generated by exposure SCLC to cisplatin similar to SR-2. SCLCBC exhibits 10 fold resistances to cisplatin, and 10 fold resistances to carboplatin but not to oxaliplatin.

*SCLCL *was established from the pleural effusion of a SCLC patient who relapsed on VP-16 and cisplatin. Unlike SCLC1 and SCLCB, this cell line grows as a floating aggregate. SCLCL does not overexpress P-gp 1 or MRP1 and is positive for chromogranin.

*NSCLCG *was established from a chest wall mass of a patient with adenocarcinoma of the lung who failed cisplatin and taxotere. This cell line is positive for keratin and does not overexpress P-gp1 and MRP1.

*NSCLCW *was established from the pleural effusion of a patient with poorly differentiated adenocarcinoma of the lung who failed gemcitabine + cisplatin, taxol + carboplatin and irinotecan + oxaliplatin. This cell line does not overexpress P-gp1 or MRP1.

All cell lines were maintained on RPMI supplemented with 10% FBS.

### Compounds

CCI-779 and rapamycin were kindly provided by Wyerh-Ayrest. Cisplatin, carboplatin, oxaliplatin, VP-16, doxorubicin and vinblastine were obtained from the hospital pharmacy.

### Growth Inhibitory effect

1 × 106 cells were seeded onto 24-well plates and allowed 8 hr. for attachment. Various concentrations of rapamycin or its analogs CCI-779 or other chemotherapeutic drugs listed above were added to each well. Each concentration was performed in duplicate. After 72 hr. exposure, viable cells were counted in the presence of 0.2% trypan blue. The growth inhibitory effect (ID50) was determined by plotting the number of viable cells as a percentage of control against the compound concentration[26]. The growth inhibitory effect was performed after cells were kept in drug free media for 14 days.

### Assay for Apoptosis by Annexin V

Cells were treated with cisplatin with and without CCI-779 for 24 hrs. and then assay for apoptosis using Annexin V-FITC Apoptosis Detection kit (BD Bioscience). The kit includes annexin V conjugated to FITC and propidium iodide. For each sample, 10^5 ^or 10^6 ^cells were collected, washed in PBS and gently suspended in 100 μL of binding buffer (10 mM HEPES pH 7.4, 150 mM NaCl, 5 mM KCl, 1 mM MgCl_2_, 1.8 mM CaCl_2_) containing 0.25 μg annexin V-FTIC. Incubation lasted 15 min in the dark at room temperature. Finally, cells were washed and suspended in the binding buffer with 5 μg propidium iodide and analyzed with Coulter XL flow cytometer. At least 10,000 cells were counted per analysis.

### Westernblot Analysis of mTOR

Cells were lysed with RIPA buffer (10 mMTris pH7.4 100 mM NaCl, 1 mM EDTA, 20 mM Na_4_P_2_O_7_, 2 mM Na_3_VO_4 _10%, 0.5% deoxycholate, 1 mM PMSF) and protease inhibitor cocktail from Sigma and passed several times through a 23G needle, and centrifuged. The total protein was separated on 8% SDS-PAGE, transferred onto membrane and immunoblot with rabbit polyclonal antibody to mTOR (purchased from Upstate) and detected by chemiluminescence.

### Westernblot Analysis of 4E-BP

The method described by Gingra et.al. was used [27]. Cells were seeded at 1 × 10^5 ^cells /ml onto 100 mm dishes, allowed overnight for attachment, then treated with various doses of cisplatin, with and without CCI-779. At 24 hrs, cells were harvested by scraping with RIPA buffer and protease inhibitor cocktail (purchased from Sigma). Lysis was completed by passing the cells through a 25 G needle. To bind elF-4E, 25 ul of 7methyl-GTP Sepharose was added to the lysates and incubated overnight at 4°C. The complexes were collected by centrifuge, washed with lysis buffer, then dissociated from sepharose by adding 50 ul of SDS-PAGE loading buffer, heated to 95°C then separated by SDS-PAGE, probed with 4E-BP antibody (purchased from Cell Signaling) and detected by chemiluminescence.

### Westernblot Analysis for p70-S6 Kinase

This is similar to analyses of 4E-BP. Cells were seeded at 1 × 10^5 ^onto 100 mm dishes, exposed to cisplatin alone or in combination with CCI-779 overnight, lysed with RIPA buffer and then separated by SDS-PAGE. The membrane was probed with phospho-p70-S6 kinase antibody purchased from Cell Signaling (phospho-thre.389) and detected by chemiluminescence. This is a mouse monoclonal antibody which recognizes phospho-p70 S6 kinase.

### Westernblot Analysis for elongation factor α

Cisplatin resistant cells (SR-2 and SCLCBC) were treated with cisplatin or CCI-779 or combination of CCI-779 and cisplatin for 24 hours. Protein extracts were obtained and separated by SDS-PAGE and probed with anti-EF-1α monoclonal antibody (purchased from Upstate) and detected with chemiluminescence. This antibody detects a 53 kDa protein.

## Results

### Growth Inhibitory Effect of rapamycin and CCI-779

Table 1 shows the result of growth inhibitory effect of rapamycin and CCI-779 in a panel of parental and resistant SCLC lines. Both CCI-779 and rapamycin are equally active in parental cell lines as well as their cisplatin resistant variants. Furthermore, CCI-779 is also active in cell lines which were established from patients who failed cisplatin containing regimens with the ID50 ranging from 0.1–0.5 ug/ml. In contrast, CCI-779 and rapamycin have no activity in P-gp1 or MRP1 overexpressing cell lines with the ID50 of 6.9 and 7.5 ug/ml, respectively. This is not surprising, since the structure of rapamycin suggest that it may be a P-gp substrate [28].

**Table 1:** Growth inhibitory effect of Rapamycin and CCI-779 (ID_50_)

	SCLC1	SR-2	SCLCB	SCLCBC	SCLCL	SCLCR	SCLCA	NSCLCW	NSCLCG
Rapamycin ug/ml	0.66 ± 0.1	0.1 ± .0.05	0.05 ± 0.02	0.09 ± 0.03	0.2 ± 0.006	>1	>1	ND*	ND*
CCI-779 ug/ml	0.07 ± 0.02	0.06 ± 0.01	0.04 ± ..0.01	0.05 ± 0.02	0.3 ± 0.1	6.9 ± 2.4	7.5 ± 1.5	0.3 ± 0.1	0.5 ± 0.2

*ND = not done. The ID_50 _represent the average of two experiments, each experiment done in duplicate.

### Growth Inhibitory effect of CCI-779 in combination with cisplatin

Since CCI-779 is equally or slightly better than rapamycin in terms of growth inhibitory effects and is currently in clinical trial, we have further investigated whether the addition of CCI-779 can indeed increase cisplatin sensitivity, We have studied growth inhibitory effect of cisplatin in the presence of 10 ng/ml of CCI-779. At this dosage, the growth inhibitory effect is less than 10%. The results are shown in table 2. CCI-779 is able to reduce the ID50 of cisplatin to 3–6 times in all cisplatin resistant cell lines. However, CCI-779 does not alter the ID50 in the parental line. Thus, it appears that CCI-779 can restore cisplatin sensitivity in otherwise cisplatin resistant cells. We further investigated whether CCI-779 can completely reverse cisplatin resistance. SR.2 and SCLCBC were treated with the cisplatin ID50 of sensitive cells (0.15 ug/ml in SCLC1 and 0.25 ug/ml in SCLCBC) and various concentrations of CCI-779. Our results indicate that CCI-779 at 0.03 ug/ml can completely reverse cisplatin resistance in both SR-2 and SCLCBC.

**Table 2:** Growth inhibitory effect of Cisplatin (ID_50_) with and without CCI-779

	Cisplatin (ug/ml)	Cisplatin (ug/ml)+ CCI-779
SCLC1	0.15 ± 0.05	0.18 ± 0.02
SR-2	2.52 ± 0.51	0.45 ± 0.03
SCLCB	0.25 ± 0.12	0.14 ± 0.02
SCLCBC	2.57 ± 0.23	0.55 ± 0.04
SCLCL	0.95 ± 0.08	0.3 0 ± 0. 05
NSCLCW	1.2 ± 0.07	0.28 ± 0.04
NSCLCG	1.5 ± 0.5	0.4 ± 0.07

**Figure 1:**
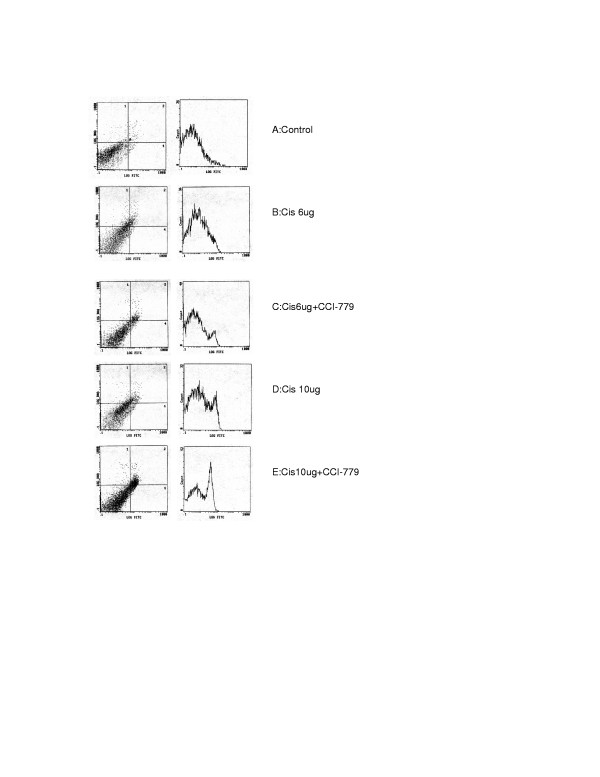
Apoptosis assay using annexin-V by FACS analysis in cisplatin resistant cell line (SR-2). Cells were treated with cisplatin alone or in combination with 0.15 ug of CCI-779. Left panels are dual staining for propidium iodide uptake and annexin-V^FITC^, Right panels are corresponding distribution of annexin V^FITC ^staining in populations of cells. 1A: control. 1B: SR-2 treated with 6 ug of cisplatin (No annexin V^FITC ^was detected) 1C: SR-2 treated with 6 ug of cisplatin+CCI-779 (small peak of annexin V^FITC ^was detected) 1D: SR-2 treated with 10 ug of cisplatin (a peak of annexin V^FITC ^was detected) 1E: SR-2 treated with 10 ug of cisplatin+CCI-.779 (a large peak of annexin V^FITC ^was detected) Note: CCI-779 alone at 0.15 and 0.3 ug/ml did not induce apoptosis (data not shown).

We have further investigated whether combination of CCI-779 with cisplatin can also induce apoptosis in cisplatin resistant cells. The results are shown in fig 1. Cisplatin at 6 ug/ml cannot induce apoptosis in these resistant cells, while at a higher dose (10 ug/ml), the apoptotic effect is small but discernable. However, when CCI-779 (150 ng) was added to cisplatin, the effect is seen at 6 ug/ml of cisplatin and increases at 10 ug/ml. Thus, our data clearly shows that the addition of CCI-779 to cisplatin in these resistant cells not only augments the growth inhibitory effect, but also the apoptotic effect of cisplatin. Similar results were also obtained with SCLCBC cell lines. It is noteworthy that, CCI-779 alone at 300 ng/ml did not induce apoptosis (data not shown) in both cisplatin sensitive cells (SCLC1 and SCLCB).

### The effect of CCI-779 in reversing doxorubicin resistance in P-gp1 and MRP1 overexpressing cells

It has been shown that rapamycin can block P-gp efflux pump [15,16], but is less potent than cyclosporin. We have studied whether CCI-779 can block P-gp and MRP1 efflux pump and consequently increased doxorubicin sensitivity in these P-gp1 and MRP1 overexpressing cells. Our data demonstrated that at 10 ng/ml of CCI-779, there is no effect on the ID50 of doxorubicin in both P-gp1 and MRP1 overexpressing cells. However, at 1 ug/ml CCI-779 is able to decrease the ID50 from 0.04 to 0.009 ug/ml. The data are summarized in table 3. Thus, our data suggest that CCI-779 is a weak P-gp blocker compared to those previously reported for rapamycin.

**Table 3:** Growth inhibitory effect of Doxorubicin (ID_50_) with and without CCI-779

	DOX (ug/ml)	DOX+0.01 ug CCI-779	DOX+1 ug CCI-779
SCLCA	0.09 ± 0.02	0.07 ± 0.01	0.08 ± 0.02
SCLCR	0.042 ± 0.01	0.058 ± 0.02	0.009 ± 0.005

### MTOR expression by westernblot analysis

**Figure 2:**
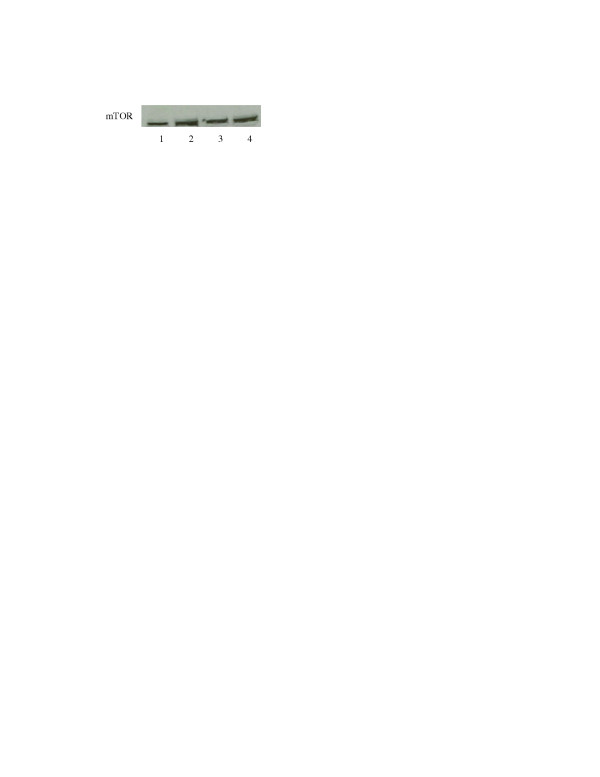
Westernblot analysis of mTOR in Lane1: SCLCB, Lane 2: SCLCBC. Lane 3: SR-2, Lane 4: SCLC1. Note: there are no differences in mTOR levels in these 4 cell lines.

It is known that mTOR is a target of CCI-779. It is possible that sensitivity to mTOR inhibitor may be related to mTOR levels. We have studied mTOR expression in the cisplatin sensitive and resistant cells. The result is shown in fig 2. There are no discernable differences in mTOR levels in these cell lines.

### 4E-BP phosphorylation

**Figure 3:**
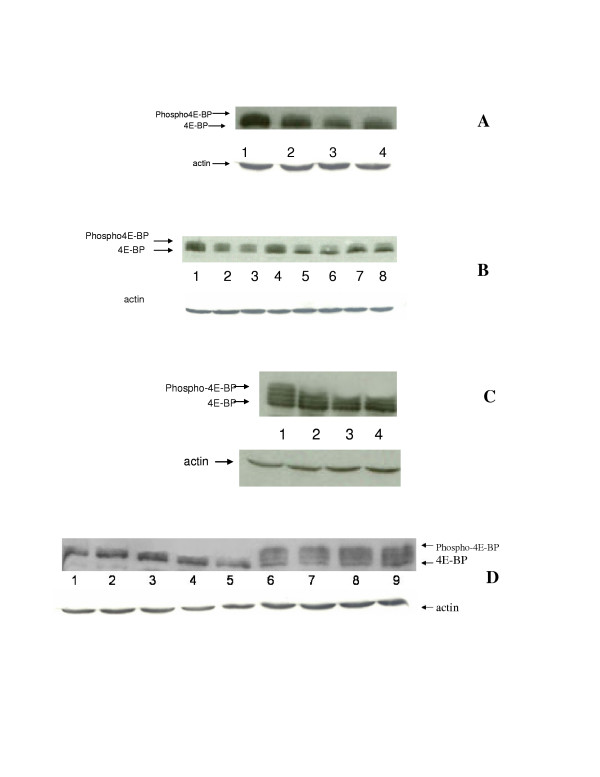
Westernblot analysis of 4E-BP phosphorylation in parental and cisplatin resistant small cell lung cancer cell lines. A: SCLC1 (parental line) was treated with 0.1 ug/ml of cisplatin alone, CCI-779 alone or in combination. Lane1: control (untreated cells) which showed high amount of phosphorylated 4E-BP. Lane 2: SCLC1 treated with 0.1 ug/ml of cisplatin which showed a decrease in the amount of phosphorylated 4E-BP, lane 3&4: SCLC1 treated with 150 ng/ml of CCI-779 alone and CCI-779 with cisplatin 0.1 ug/ml respectively. Both showed comparable amount of phosphorylated 4E-BP and are less than control cells and or cells treated with cisplatin alone. B: 4E-BP phosphorylation in SR-2 (cisplatin resistant cell line derived from SCLC1) after treatment with cisplatin with and without CCI-779. Lane 1: Control untreated cells which showed an abundant amount of phospho-4E-BP, lane 2, 3, and 4: SR-2 treated with 5,10 and 0.1 ug/ml of cisplatin. The amount of phospho-4E-BP are not affected by cisplatin at low dose, but slightly decreased at high dose. Lane 5,6 SR-2 treated with CCI-779 at 150 ng and 300 ng/ml respectively. Predominantly unphosphorylated 4E-BP is seen, Lane 7&8: SR-2 treated with 150 ng/ml of CCI-779 combined with cisplatin 5 ug and 10 ug/ml respectively. Small amounts of phosphorylated 4E-BP are seen and much less than those with cisplatin alone (lane 2,3&4). C:4E-BP phosphorylation in SCLCB(parental line). Lane 1: Control untreated cells which showed an abundant amount of phospho-4E-BP, lane 2: SCLCB treated with 0.1 ug/ml of cisplatin showed a decrease amount of phospho-4E-BP. lane 3 & 4 SCLCB treated with 150 ng/ml of CCI-779 alone and CCI-779 150 ng/ml with 0.1 ug/ml of cisplatin respectively. Both showed comparable amount of phospho-4E-BP and is slightly less than cisplatin alone. D: 4E-BP phosphorylation in SCLCBC (cisplatin resistant cells). Lane 1,2,3: SCLCBC treated with CCI-779 150 ng/ml combined with cisplatin at 1,5 and 10 ug/ml of cisplatin respectively. The amount of phospho-4E-BP is minimum and predominantly unphosphorylated form, lane 4&5 SCLCBC treated with CCI-779 at 150 ng and 300 ng/ml, only unphosphorylated 4E-BP predominate. Lane 6,7,8 SCLCBC treated with 10,5 and 1 ug/ml of cisplatin respectively. The amount of phospho4E-BP are similar to the control cells. Lane 9. Control untreated cells. Actin is used as control for protein loading for all cell lines

It is well known that mTOR phosphorylates 4E-BP and releases its binding from eIF-4E. This results in translation initiation. Inhibition of mTOR will prohibit its ability to phosphorylate 4E-BP and thereby inhibit the translation process. We have studied 4E-BP phosphorylation in SCLC1/SR2, SCLCB/SCLCBC. Our results are shown in fig 3. Cisplatin does not affect 4E-BP phosphorylation in cisplatin resistant cell lines. However, the addition of CCI-779 inhibits this process. We have tested two different doses of CCI-779 (150, and 300 ng/ml), our data indicate that 150 ug/ml is as efficient as 300 ug/ml in inhibiting 4E-BP phosphorylation. We have further reduced the dose to 100 ng and the effect on 4E-BP phosphorylation are similar to 150 ng/ml(data not shown). In contrast, in sensitive cells, the phosphorylation of 4E-BP decreased after the addition of cisplatin alone (fig 3, panel A&C). This effect is only slightly enhanced by CCI-779. These results correspond with the growth inhibitory effect illustrated in table 2 which showed that CCI-779 decreased the ID50 in cisplatin resistant cells but has only minimal effect on sensitive cells.

### P-70S6 kinase phosphorylation

**Figure 4:**
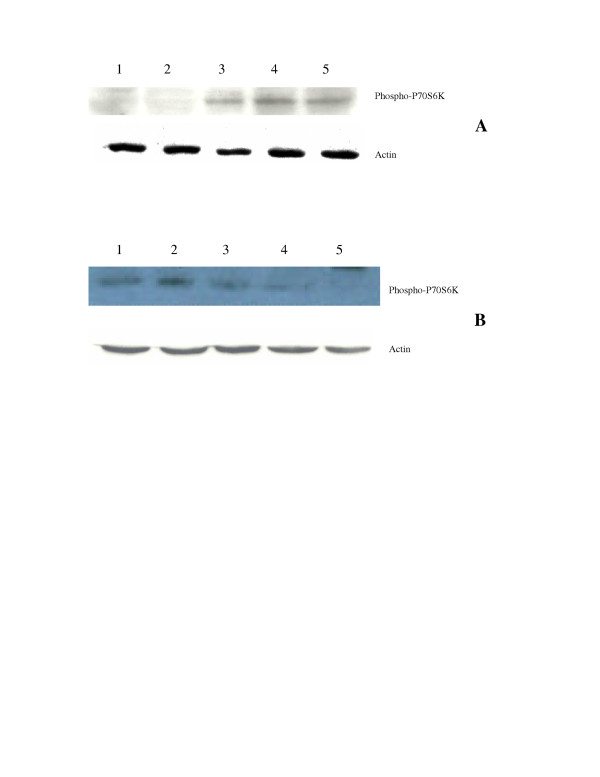
Westernblot analysis of phospho-P70S6kinase in cisplatin resistant cells. A: SR-2 cells. Lane 1. SR-2 treated with cisplatin 10 ug/ml and CCI-779 150 ng/ml, lane 2: SR-2 treated with CCI-779 at 150 ng/ml. Both 1&2 showed only minimal amount of phospho-p70S6kinase. Lane 3&4 SR-2 treated with cisplatin at 0.1 and 10 ug/ml., cisplatin does not affect the amount of phospho-p70S6kinase and showed similar intensity to control cells. Lane 5: control untreated cells. B. SCLCBC cells. Lane 1: Control untreated cells, lane 2&3 SCLCBC treated with cisplatin at 0.1 and 10 ug/ml respectively. Similar amounts of phospho-p70S6kinase were seen. Lane 4 SCLCBC treated with cisplatin 10 ug/ml and CCI-779 150 ng/ml. The amount of phosphor-p70S6kinase is less. Lane 5 SCLCBC treated with CCI-779 at 150 ng/ml. No signal intensity for p-70S6kinase was seen. Actin was used as control for protein loading.

It is well known that CCI-779 decreases p-70 S6 kinase activity by inhibiting its phosphorylation.. We have determined the levels phospho-P-70 S6 kinase by westernblot analysis. Our results are shown in fig 4. Cisplatin at 0.1 ug/ml or 10 ug/ml did not affect the phosphorylation in two cisplatin resistant cell lines, however, the phosphorylation decreased after adding CCI-779. In contrast, in parental cells (SCLC1 and SCLCB), the levels of phospho-p-70 S6 kinase is decreased after adding cisplatin at 0.1 ug/ml and decreased slightly after the addition of CCI-779(data not shown). These findings are similar to the phosphorylation of 4E-BP.

### Assay for elongation factor alpha (eEF1-α)

**Figure 5:**
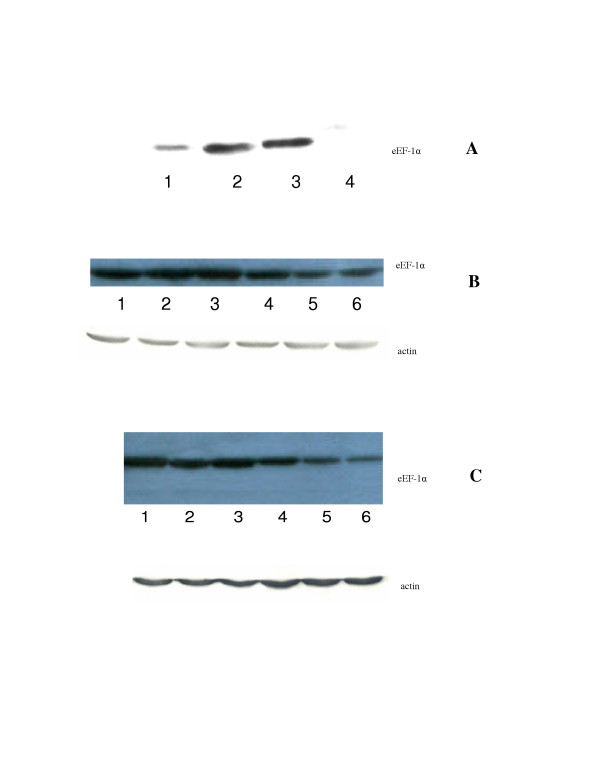
Westernblot analysis of eEF-1α. **A **eEF-1α in sensitive and resistant cells. Lane 1. SCLCB, lane 2: SCLCBC, lane 3: SR-2, lane 4: SCLC1. The signal intensity of the resistant cell line SCLCBC and SR-2 are much greater than their sensitive cells SCLCB and SCLC1 counterpart. **B **eEF-1α in SR-2 cell line. Lane 1: control, lane 2&3: SR-2 treated with 0.5 and 10 ug/ml of cisplatin respectively. Similar signal intensity are seen compared to control, lane 4&5: SR-2 treated with 10 ng and 50 ng/ml of CCI-779 respectively. There is no change in signal intensity of eEF-1α at 10 ng of CCI-779, but decreased after exposure to 50 ng/ml of CCI-779, lane 6: SR-2 treated with 10 ug/.ml of cisplatin with CCI-779 50 ng/ml, the signal intensity also less and similar to those treated with CCI-779 alone. **C **eEF-1α in SCLCBC. Lane 1: control, lane 2&3 SCLCBC treated with 0.5 ug and 10 ug/ml of cisplatin respectively. Similar signal intensity of eEF-1α is seen compared to control, lane 4&5: SR-2 treated with 10 ng and 50 ng/ml of CCI-779 respectively, there is no changes in signal intensity at 10 ng/ml of CCI-779, but decreased at 50 ng/ml of CCI-779, lane 6 SCLCBC treated with 10 ug/ml of cisplatin with CCI-779 50 ng/ml. The signal intensity is less and similar to those treated with CCI-779 alone.

It has been shown that rapamycin can selectively inhibit translation of mRNA encoding elongation factor alpha. In addition, our cisplatin resistant cell lines also overexpress elongation factor α (fig 5, panel A). We have examined whether CCI-779 has effect on elongation factor alpha. The data are shown in fig 5 (panel B&C). Cisplatin at 10 ug/ml has no effect on eEF-1 α in these resistant cell lines. However, after adding CCI-779, the eEF-1 α is much less.

## Discussion

The mechanisms of cisplatin resistance have been studied for the last several decades. To date, there is no effective pharmacological manipulation to overcome this form of complex resistance. In this report, we have found that mTOR inhibitor (CCI-779) is able to restore cisplatin sensitivity. This is most likely due to the fact that mTOR inhibitor can down regulate protein synthesis. mTOR regulates translation of proteins which are involved in cell cycle progression and cell growth [12,29-32]. The two best characterized downstream targets of mTOR are two families of proteins that control translation, the ribosomal protein S6 kinase (S6K) and the eukaryotic initiation factor eIF4E binding protein (4E-BP)[12,33-35]. S6K1 (p70S6 kinase) phosphorylates S6 which controls the translation of mRNA that possesses an unusual oligopyrimidine tract at its transcription starting site termed 5'TOP. These 5'TOP mRNAs encode major components of protein synthesis machinery (ribosomal protein and elongation factor). Another downstream target of mTOR is eLF-4E binding protein 4E-BP. eIF-4E is known to control translation of mRNA with secondary structure (often GC rich)[36]. These mRNAs are known to encode proteins related to proliferation. In this study, we have shown that in cisplatin resistant cells the phosphorylation of 4E-BP is not affected by cisplatin alone, but decreased after adding CCI-779 which indicates that CCI-779 can block the translational initiation. Similar results were obtained for the phosphorylation of p-70 S6 kinase. In contrast, in parental (cisplatin sensitive cells), cisplatin alone is able to decrease the phosphorylation of both 4E-BP and p-70S6 kinase, although this effect is slightly augmented with the addition of CCI-779. These results correspond with our growth inhibitory findings which show that the addition of CCI-779 only slightly augments the growth inhibitory effect of cisplatin in sensitive cells. The ability of cisplatin to inhibit 4E-BP and p-70 S6 kinase in cisplatin sensitive cells has been reported by others [37]. The exact mechanism is not known. Nevertheless, this mode of action appears to diminish in cisplatin resistant cell lines, and these cells are able to readily turn on the translational machinery. Overall, our findings support our notion that the reason why cisplatin resistant cell lines up regulated the mRNA encoding for ribosomal protein and elongation factors is due to the fact that these resistant cells have to turn on the protein synthesis machinery in order to survive. Consequently, by inhibiting the protein translational machinery using CCI-779, cisplatin resistance can be overcome. Furthermore, our preliminary results also show that CCI-779 can inhibit the synthesis of DNA repair proteins, cell cycle protein as well as telomerase which further support our hypothesis. Importantly, these findings also can be applied to de-novo cisplatin resistant cell lines derived from patients who failed treatment (SCLCL, NSCLCW and NSCLCG). The ID50 of cisplatin was decreased by 3–4 folds after CCI-779. The concentration we used to overcome cisplatin resistance is low (10 ng/ml) and is clinically achievable. [38,39] These findings can have significant clinical implication for future use of mTOR inhibitors to overcome cisplatin resistance.

It has been previously demonstrated that rapamycin can reverse MDR1 mediated drug resistance [15]. Furthermore, both P-gp1 and MRP1 mediated drug resistance have been reported in lung cancer [40-42]. Thus, we have also investigated the possible antitumor activity as well as reversal activity of CCI-779 in P-gp1 and MDR1 overexpressing cell lines. Our results demonstrated that both CCI-779 and rapamycin have no antitumor activity in P-gp1 or MRP1 overexpressing cell lines. It is possible that both rapamycin and CCI-779 are recognized by both P-gp1 and MRP1. This is not surprising since it has been reported that rapamycin can be transported by P-gp in renal proximal tubule [43]. We have also found that CCI-779 is a weak reversal agent when compared with those reported for rapamycin. CCI-779 at 1 ug/ml (1 uM) can only decrease the ID50 of doxorubicin by 4.5 fold whereas rapamycin at 1 uM can decrease the ID50 of daunorubicin by 10 fold [15]. This may be due to the fact that CCI-779 is more water soluble and thus is less effective as reversal agent [44]

## Conclusion

We conclude that CCI-779 is able to restore cisplatin sensitivity in small cell lung cancer cell lines which were selected for cisplatin resistance aswell as cell lines derived from patients who failed cisplatin. The most likely mechanism is due to inhibition of translation of proteins which are involved in cisplatin resistance. These findings should be further explored in the clinic. However, CCI-779 has no antitumor activity in P-gp1 and MRP1 overexpressing cell lines. Moreover, CCI-779 can only partially reverse P-gp1 at a higher dose (1 ug/ml). Therefore, CCI-779 is not a good candidate to use in MDR1 or MRP1 overexpressing cells.
